# Rapid Discrimination of the Geographical Origins of an Oolong Tea (Anxi-Tieguanyin) by Near-Infrared Spectroscopy and Partial Least Squares Discriminant Analysis

**DOI:** 10.1155/2014/704971

**Published:** 2014-06-26

**Authors:** Si-Min Yan, Jun-Ping Liu, Lu Xu, Xian-Shu Fu, Hai-Feng Cui, Zhen-Yu Yun, Xiao-Ping Yu, Zi-Hong Ye

**Affiliations:** ^1^Zhejiang Provincial Key Laboratory of Biometrology and Inspection and Quarantine, College of Life Sciences, China Jiliang University, Hangzhou, Zhejiang 310018, China; ^2^China National Institute of Standardization, Beijing 100191, China

## Abstract

This paper focuses on a rapid and nondestructive way to discriminate the geographical origin of Anxi-Tieguanyin tea by near-infrared (NIR) spectroscopy and chemometrics. 450 representative samples were collected from Anxi County, the original producing area of Tieguanyin tea, and another 120 Tieguanyin samples with similar appearance were collected from unprotected producing areas in China. All these samples were measured by NIR. The Stahel-Donoho estimates (SDE) outlyingness diagnosis was used to remove the outliers. Partial least squares discriminant analysis (PLSDA) was performed to develop a classification model and predict the authenticity of unknown objects. To improve the sensitivity and specificity of classification, the raw data was preprocessed to reduce unwanted spectral variations by standard normal variate (SNV) transformation, taking second-order derivatives (D2) spectra, and smoothing. As the best model, the sensitivity and specificity reached 0.931 and 1.000 with SNV spectra. Combination of NIR spectrometry and statistical model selection can provide an effective and rapid method to discriminate the geographical producing area of Anxi-Tieguanyin.

## 1. Introduction

Oolong tea has been one of the most popular traditional beverages in the world. As a semifermented tea, Oolong tea is somewhere between the green and black tea with pleasurable aroma and taste for its particular processing. Moreover, a lot of people regard oolong tea as a functional drink for its high content of antioxidative substracts like epigallocatechin gallate (EGCG) and catechins [1]. And oolong tea is reported to have effect on antiobesity, preventing decayed tooth, disinfecting and hypolipidemic actions [2, 3].

The special aroma and taste of a tea depend largely on geographical and natural conditions of tea tree growing as well as on tea cultivar, cultivation traditions, and processing procedures. Therefore, most of the famous teas in China are named after their origins. Anxi-Tieguanyin tea (ATT) is one of the most famous Oolong teas with protected geographical indication (PGI), produced from Anxi County, a small town in Fujian province. Due to individual climate and edatope in Anxi County, ATT has a long-lasting fragrance and a strong aftertaste. It has been exported to many places all over the world. As the Tieguanyin tea with different provenances has similar appearance, some merchants fraudulently label ATT indication to non-Anxi-Tieguanyin teas (NATT) for illegal profits [4]. These actions do damage the reputation of ATT [5]. Therefore, it is important and urgent to employ quality control of ATT against various counterfeits. Until now, sensory analysis is the usually used method in distinguishing specific ATT geographical origin, which depends basically on the experience and personal emotion of tea tasters. For this application, a more stable and effective tool is worthwhile and necessary to be developed.

Different cultivating places had varied growing conditions including altitude, climate, soil, microelement, fertilizer, and processing [6, 7]. All these factors contribute to the different chemical components in teas. Although varied pattern of several chemical components could partially indicate the characters of specific teas, making errorless discriminations just by several chemical components proved difficult because the components in teas are really complicated [8, 9]. In recent years, instrumental methods coupled with chemometrics have provided promising alternative approaches in food components analysis [10–12]. As one of the rapid and effective measuring instruments, the near-infrared (NIR) spectroscopy has been widely performed in food multivariate quality control [13, 14]. Depending on individual vibrational frequency of molecular structure, NIR could characterize multiple chemical components of samples, which can help researchers discriminate the provenances of tea products. NIR has the following advantages over chemical analysis: (1) less money and time cost in analysis; (2) being nondestructive for samples; and (3) the ability in online analysis [15].

For automatic identification, some researchers have successfully used NIR spectroscopy and class models to discriminate the provenances of green teas [16, 17]. However, as a semifermented tea, the chemical components in Tieguanyin tea are far more complicated; it requires higher sensitivity in measuring process and classification. This paper aims to provide an effective way to discriminate the geographical origin of ATT by NIR spectroscopy and PLADA.

## 2. Materials and Methods 

### 2.1. Tea Samples

450 authentic ATT samples were collected from 30 main Tieguanyin-producing areas of Anxi County with official certifications. 120 NATT samples were collected from Yongchun, Huaan, Xiandu, and so forth. All these samples were spring teas of 2013 (bought in the local tea markets of Anxi County before May 23, 2013) and were preserved in cold storage (4°C) before measuring analysis. The detailed information concerning samples was presented in Table 1.

### 2.2. NIR Spectrometric Analysis

All of the samples were scanned by TENSOR37 Fourier transform NIR spectrometer (Bruker, Ettlingen, Germany) and OPUS 7.2 software. Each sample was packed in a quartz cuvette and detected with a PbS detector. Each reported spectrum is the average of 64 scanning spectra in the spectral range from 4000 cm^−1^ to 12000 cm^−1^. Here the resolution was 8 cm^−1^ and the scanning interval was 1.928 cm^−1^, so 4148 individual data points were acquired from each spectrum for multivariate analysis.

### 2.3. Data Preprocessing and Splitting

Data analysis is performed on MATLAB 7.14.0.739 (Mathworks, Sherborn, MA). Aberrant spectra (outliers) are usually caused by abnormal samples or measuring faults. For class models, outliers make negative influence, sometimes even leading to model bias. Therefore, the Stahel-Donoho estimates (SDE) were used to detect abnormal spectra. SDE can detect the multiple outliers by calculating the values of outlyingness [18]. In addition, to ensure the sensitivity and the specificity, spectra need to be preprocessed. Three preprocessing methods were investigated in this study, including standard normal variate (SNV) transformation [19], taking second-order derivatives (D2), and smoothing [20].

With the spectra preprocessed, the data were split into a training set and a prediction set by the Kennard and Stone (K-S) algorithm [21]. K-S algorithm can ensure that the prediction objects are uniformly distributed in the range of training objects. In this paper, K-S algorithm was performed separately for ATT and NATT samples. Then two training sets were put together as a total training set and two prediction sets formed a total prediction set.

### 2.4. PLSDA

As a key method in chemometrics, partial least square has various applications [22–24]. The spectra of training set can be represented as an *n* × *p* matrix *X* (with *n* training objects and *p* wavelength points). *s* is the value of sorted number, in this paper, *s* = 2 (the ATT class and the NATT class), and an *n* × *s* matrix *Y* is then designed. The value of each element in *Y* is the corresponding category of the object in *X*. If an object *i* (*i* = 1 : *n*) is from class *j* (*j* = 1 : *s*), then element at *i*th row and *j*th column in *Y* is given a value 1; all other elements in *Y* are set −1. For prediction, a new sample is classified into class *j* (*j* = 1 : *s*) when the *j*th element of its predicted response is above zero. Because *s* = 2, we just need to consider the first column of predicted responses. If the value of the first column is above zero, it will be classified into ATT class; otherwise, it is NATT class. For PLSDA, the number of latent variables is a key parameter. Too many latent variables will cause the risk of overfitting. So in this model, Monte Carlo cross-validation (MCCV) [25] was performed to estimate the parameter. The number of latent variables was selected to calculate the minimal misclassification rate of MCCV (MRMCCV):

(1) $$\begin{array}{c} \text{MRMCCV}=\left(\overset{T}{\underset{i=1}{\sum }}\frac{M_{i}}{P_{i}}\right)\times 100\%, \end{array}$$

where *M*_*i*_ is the number of misclassified objects, *P*_*i*_ is the total number of prediction objects, and *T* is the times of data splitting; then sensitivity and specificity were calculated to evaluate the performance of classification models:

(2) $$\begin{array}{c} \text{Sens}.=\frac{\text{TP}}{\text{TP}+{\text{FN}}^{\prime }},\\ \text{Spec.}=\frac{\text{TN}}{\text{TN}+{\text{TP}}^{\prime }}, \end{array}$$

where TP represents the number of true positives, FN is the number of false negatives, TN represents the number of true negatives, and FP is the number of false positives.

## 3. Results and Discussions

**Figure 1:**
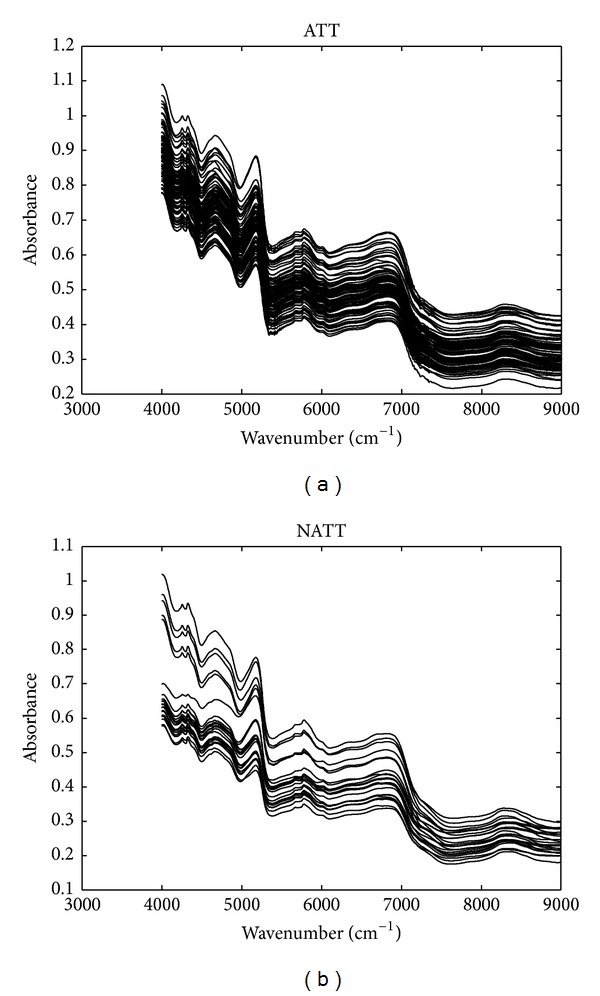
Some NIR raw spectra of Anxi-Tieguanyin (a) and non-Anxi-Tieguanyin (b) tea samples.

Some of the raw NIR spectra of ATT and NATT were demonstrated in Figure 1. The wavenumbers from 9000 to 12000 cm^−1^ were discarded in further analysis because these wavenumbers had low response signals and carried little information. Seen from Figure 1, although some of the NATT samples have lower absorbance in the range of 4000~5000 cm^−1^, a part of spectra is highly similar to the spectra of ATT and they can hardly be distinguished just by naked eyes. Therefore, to extract some useful chemical information from these spectra, chemometrics is used to develop classification models.

**Figure 2:**
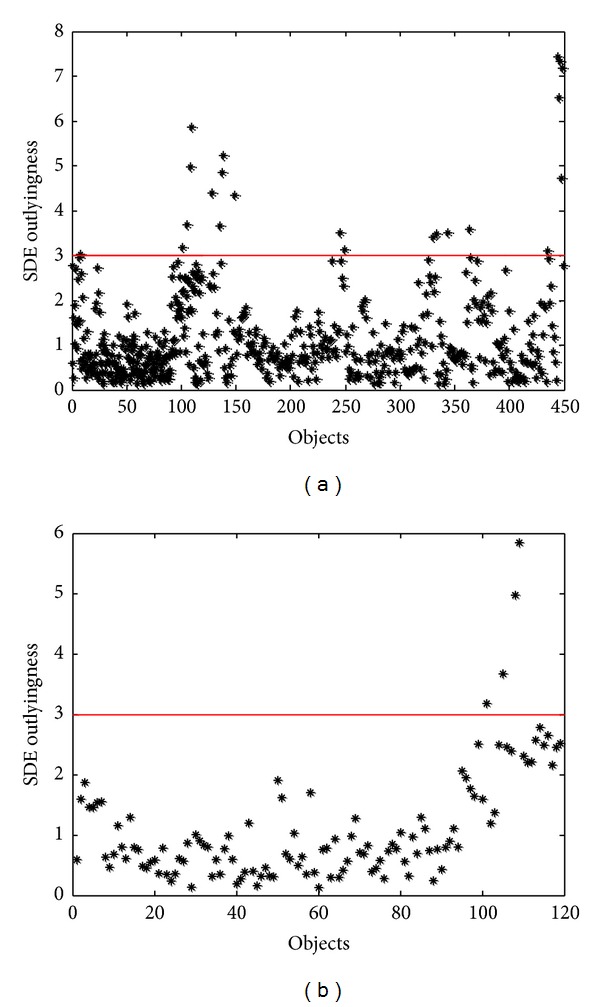
The Stahel-Donoho estimates (SDE) of outlyingness values for ATT (a) and NATT (b).

Outlier detection was performed by the SDE outlyingness diagnosis. The results were shown in Figure 2. In this paper, a spectrum is recognized as an outlier if its outlyingness is above 3. Seen from Figure 2, 19 ATT objects and 4 NATT objects were removed.

**Figure 3:**
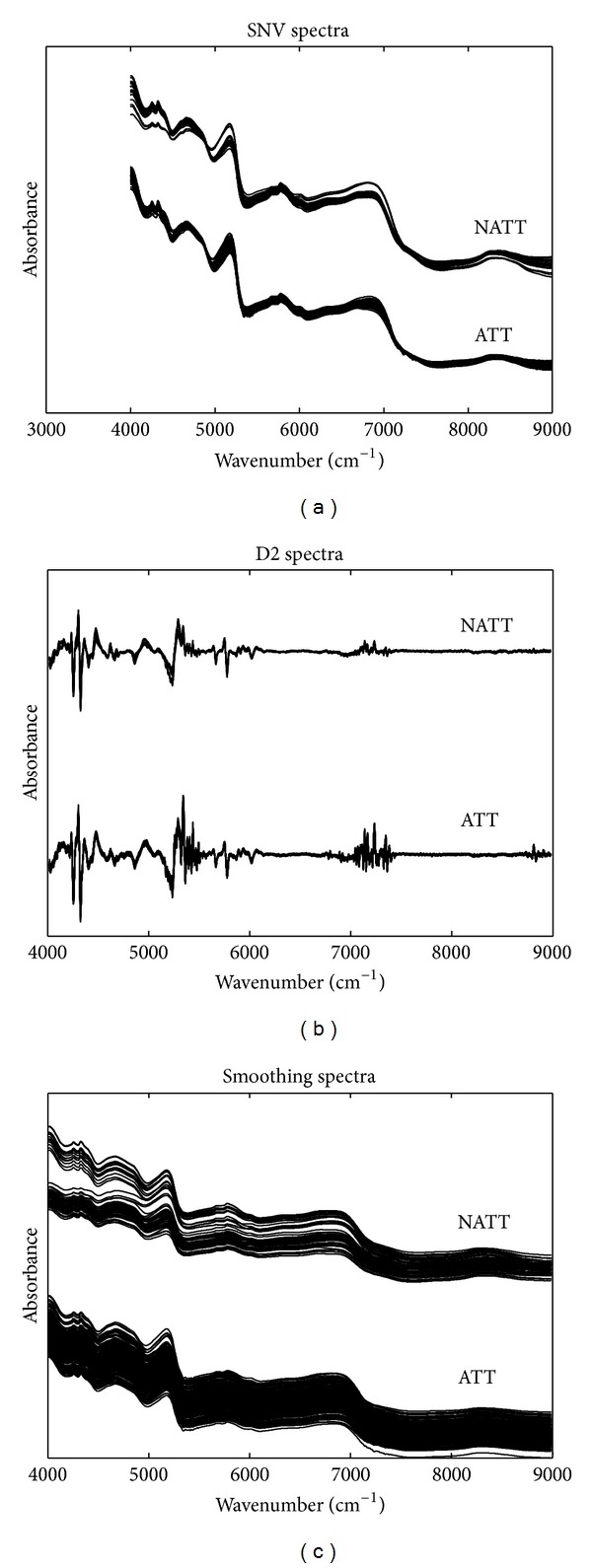
NIR spectra of samples preprocessed by SNV (a), D2 (b), and smoothing (c); an artificial shift was added to distinguish ATT and NATT.

Then the raw spectral data was preprocessed by SNV, D2, and smoothing. Preprocessed spectra were shown in Figure 3. Compared with the raw spectra, SNV can reduce some spectral variations, D2 can enhance the resolution of some bands and remove most of the baselines, and smoothing can reduce the strength of noise signals.

**Figure 4:**
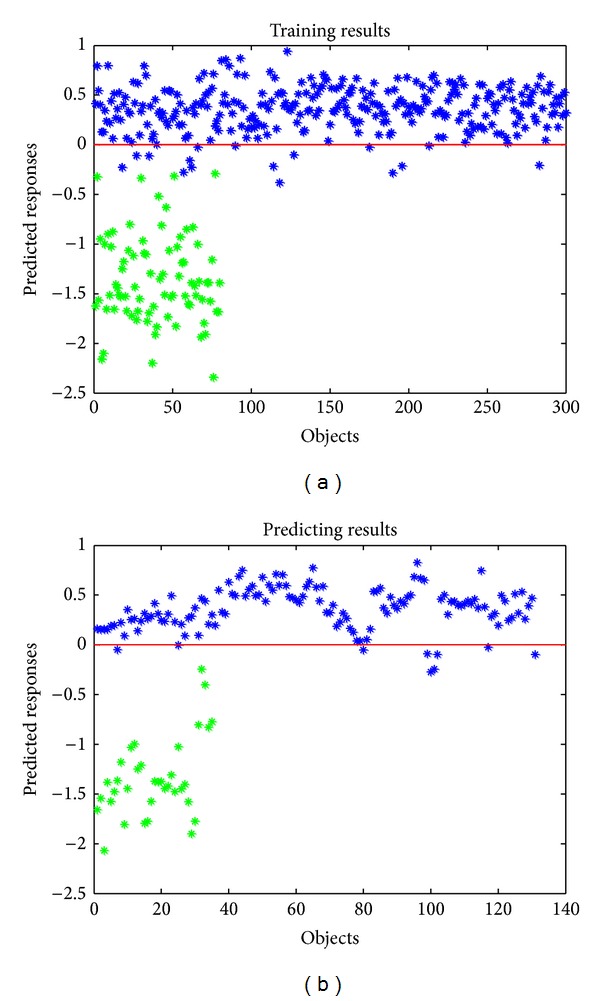
Training (a) and predicting (b) results of PLSDA model based on NIR spectra with SNV preprocessed. Blue asterisks represent the ATT objects; green asterisks represent the NATT objects.

For PLSDA, K-S algorithm was used to split the 431 ATT samples and 115 ATT samples into a training set (with 300 ATT samples and 80 NATT samples) and a prediction set (with 131 ATT samples and 35 NATT samples), and then PLSDA models were developed using the raw, D2, and SNV spectra. MCCV was used to estimate the number of PLSDA latent variables; the training set was randomly divided into a secondary training set (50%) and a secondary predicting set (50%) for 20 times. The number of latent variables was selected to calculate the minimal MRMCCV. Therefore, the objects in prediction class were applied to calculate the sensitivity and specificity of PLSDA model. The prediction results and optimized parameters with different preprocessing methods were listed in Table 2. Seen from Table 2, the best model is the SNV-PLSDA with the sensitivity/specificity of 0.931/1.000. The PLSDA model based on smoothing spectra and raw NIR spectra has exactly the same results. It means smoothing had little effect on NIR spectra. However, compared with the raw data, the model based on D2 spectra even gets a lower sensitivity/specificity. It might be caused by the loss of frequency information while taking second derivatives. The training and prediction results of SNV-PLSDA were demonstrated in Figure 4.

## 4. Conclusion

The results in this paper demonstrate the feasibility of combining NIR spectroscopy and PLSDA for discriminating the geographical origin of Tieguanyin tea. The sensitivity and specificity of PLSDA model based on SNV preprocessed spectra reached 0.931 and 1.000. Compared with the traditional methods [26, 27], a NIR spectrum of sample can be acquired within a minute and PLSDA model just needs several seconds to make a prediction. Moreover, compared with other NIR-chemometrics methods [16, 17], the samples in this study were scanned by NIR without any pretreatment, like grinding or smashing, so this method is nondestructive for samples as well. For geographical identification of teas, our future work will be trying some other sensitive measuring instruments, for example, inductively coupled plasma mass spectrometry and atomic absorption spectroscopy, and then make a comprehensive comparison with NIR.

## Figures and Tables

**Table 1:** Analyzed tea samples.

Number	Producing area	Sample size^a^	Type^b^
A1	Fuqian, Anxi, Fujian	15	A
A2	Dage, Anxi, Fujian	15	A
A3	Hongyou, Anxi, Fujian	15	A
A4	Huaizhi, Anxi, Fujian	15	A
A5	Fengtian a, Anxi, Fujian	15	A
A6	Fengtian b, Anxi, Fujian	15	A
A7	Kangsui, Anxi, Fujian	15	A
A8	Taozhou, Anxi, Fujian	15	A
A9	Xiage, Anxi, Fujian	15	A
A10	Longping, Anxi, Fujian	15	A
A11	Baiye, Anxi, Fujian	15	A
A12	Longdi, Anxi, Fujian	15	A
A13	Qianlu, Anxi, Fujian	15	A
A14	Xianrong, Anxi, Fujian	15	A
A15	Hongxing, Anxi, Fujian	15	A
A16	Xianghua, Anxi, Fujian	15	A
A17	Fuyang, Anxi, Fujian	15	A
A18	Xiangdi, Anxi, Fujian	15	A
A19	Hushang, Anxi, Fujian	15	A
A20	Shanglu, Anxi, Fujian	15	A
A21	Xueshan, Anxi, Fujian	15	A
A22	Fudi, Anxi, Fujian	15	A
A23	Nanyang, Anxi, Fujian	15	A
A24	Zhentian, Anxi, Fujian	15	A
A25	Lishan, Anxi, Fujian	15	A
A26	Baodu, Anxi, Fujian	15	A
A27	Juyuan, Anxi, Fujian	15	A
A28	Huayun, Anxi, Fujian	15	A
A29	Shanling, Anxi, Fujian	15	A
A30	Jindong, Anxi, Fujian	15	A
N1	Yongchun a, Fujian	15	N
N2	Yongchun b, Fujian	15	N
N3	Huaan, Fujian	15	N
N4	Xiandu, Fujian	15	N
N5	Xinyu, Fujian	15	N
N6	Wuyuan, Jiangxi	15	N
N7	Yichun, Jiangxi	15	N
N8	Daliangshan, Sichuan	15	N

^a^The number of the tea samples from the same provenance.

^b^A: Anxi-Tieguanyin tea; N: non-Anxi-Tieguanyin tea.

**Table 2:** Predicting results obtained by PLSDA.

	Lv^a^	Sen^b^	Spe^c^
Raw data	12	0.908 (119/131)	0.971 (34/35)
SNV	12	0.931 (122/131)	1.000 (35/35)
D2	3	0.893 (117/131)	0.886 (31/35)
Smoothing	12	0.908 (119/131)	0.971 (34/35)

^a^Number of PLADA latent variables.

^b^The sensitivity of PLSDA model.

^c^The sensitivity of PLSDA model.
